# Eye-tracking glasses in face-to-face interactions: Manual versus automated assessment of areas-of-interest

**DOI:** 10.3758/s13428-021-01544-2

**Published:** 2021-03-19

**Authors:** Chiara Jongerius, T. Callemein, T. Goedemé, K. Van Beeck, J. A. Romijn, E. M. A. Smets, M. A. Hillen

**Affiliations:** 1 Amsterdam UMC, University of Amsterdam, Department of Medical Psychology, Amsterdam Public Health, Location AMC, Meibergdreef 9, 1100 DD Amsterdam, The Netherlands; 2 PSI-EAVISE, Electrical Engineering Technology (ESAT), KU Leuven, De Nayer Campus, Sint-Katelijne-Waver, Belgium; 3 Amsterdam UMC, University of Amsterdam, Department of Medicine, Amsterdam, The Netherlands

**Keywords:** Gaze behaviour, Eye-tracking glasses, Areas-of-interest, Computer vision algorithm, Pose estimation, Person re-identification

## Abstract

The assessment of gaze behaviour is essential for understanding the psychology of communication. Mobile eye-tracking glasses are useful to measure gaze behaviour during dynamic interactions. Eye-tracking data can be analysed by using manually annotated areas-of-interest. Computer vision algorithms may alternatively be used to reduce the amount of manual effort, but also the subjectivity and complexity of these analyses. Using additional re-identification (Re-ID) algorithms, different participants in the interaction can be distinguished. The aim of this study was to compare the results of manual annotation of mobile eye-tracking data with the results of a computer vision algorithm. We selected the first minute of seven randomly selected eye-tracking videos of consultations between physicians and patients in a Dutch Internal Medicine out-patient clinic. Three human annotators and a computer vision algorithm annotated mobile eye-tracking data, after which interrater reliability was assessed between the areas-of-interest annotated by the annotators and the computer vision algorithm. Additionally, we explored interrater reliability when using lengthy videos and different area-of-interest shapes. In total, we analysed more than 65 min of eye-tracking videos manually and with the algorithm. Overall, the absolute normalized difference between the manual and the algorithm annotations of face-gaze was less than 2%. Our results show high interrater agreements between human annotators and the algorithm with Cohen’s kappa ranging from 0.85 to 0.98. We conclude that computer vision algorithms produce comparable results to those of human annotators. Analyses by the algorithm are not subject to annotator fatigue or subjectivity and can therefore advance eye-tracking analyses.

## Introduction

Human gaze direction can provide information about attention and social cognition (Frischen, Bayliss, & Tipper, 2007; Itier & Batty, 2009; Pfeiffer, Vogeley, & Schilbach, 2013; Schilbach, 2015). The assessment of gaze direction, and therefore a person’s attention, during interpersonal interaction is relevant to understand the psychology of communication and natural behaviour (Jongerius, Hessels, Romijn, Smets, & Hillen, 2020; Tatler, Hayhoe, Land, & Ballard, 2011). Mobile eye-tracking glasses are increasingly used to measure gaze behaviour during face-to-face interactions (Jongerius et al., 2020). These glasses register the gaze behaviour of the person wearing them. The glasses have one forward-looking video camera on the nose bridge which captures the environment the person is looking at and infrared video cameras facing the wearer’s eyes. The infrared video cameras facing the eyes register pupil movements through a technique called pupil centre corneal reflection. These data, amongst other geometrical features of the pupil reflections, are combined to calculate the gaze direction (Tobii Pro, 2019a). The output of mobile eye-tracking registrations is often visualised as a video of the viewer’s perspective with information about the focus of people’s gaze, depicted as a dot on the video image (i.e. a 2D position (pixel) on a video screen).

Eye-tracking glasses in their current and most advanced form are more novel and, therefore, have been used less in studies of the psychology of communication compared to screen eye-tracking (Cognolato, Atzori, & Muller, 2018; Tatler et al., 2011). Mobile and screen eye-tracking differ in how gaze is registered. Screen eye-trackers can only register a person’s gaze location on a computer screen and conclusions drawn based on screen eye-tracking experiments cannot be generalized to many real-world situations (Tatler et al., 2011). When screen eye-tracking is used to understand social, dyadic interactions, this is unavoidably done in video-call-like settings (Hessels, 2020). Wearable eye-tracking glasses offer an advanced technique to capture whatever a persons’ head is directed at, meaning that gaze direction is registered while the wearer of the eye-tracking glasses is moving around in the real world (Tatler et al., 2011). Therefore, wearable eye-tracking glasses can be used in any setting and capture gaze direction of individuals acting in a dynamic world.

Because eye-tracking glasses permit freedom of movement, this technique is particularly suitable to study gaze in face-to-face interactions between two individuals (Franchak, Kretch, & Adolph, 2018; Honma, 2013; King et al., 2013; Macdonald & Tatler, 2018; Spezio, Huang, Castelli, & Adolphs, 2007; Vabalas & Freeth, 2016; M. Ye et al., 2020). . This method has been used to study eye contact in a variety of settings, e.g. to investigate the effects of autistic traits, social anxiety, amygdala impairment in face-to-face interactions (Franchak et al., 2018; Honma, 2013; King et al., 2013; Macdonald & Tatler, 2018; Spezio et al., 2007; Vabalas & Freeth, 2016; Z. Ye et al., 2012). Eye-tracking glasses have been used to study unidirectional gaze behaviour if only one interactor wears the glasses (King et al., 2013; Spezio et al., 2007; Vabalas & Freeth, 2016) or to study mutual gaze behaviour if both interactors wear the glasses (Franchak et al., 2018; Honma, 2013; Macdonald & Tatler, 2018). Currently, there is variation among studies in how mobile eye-tracking data are analysed. Some studies have used manual frame-by-frame coding (Franchak et al., 2018; Macdonald & Tatler, 2018), e.g. to register the onset and end of mutual gazing (Macdonald & Tatler, 2018). Others have used heat maps generated by eye-tracking software, illustrating with colours the data where the gaze was located the most (e.g. red for high level of gazing and green for low level of gazing) (King et al., 2013). Yet, other studies have analysed eye-tracking data by manually drawing areas-of-interest on the eye-tracking data (Vabalas & Freeth, 2016). Research using eye-tracking data would benefit from a more standardized analyses and from more efficient methods to enhance comparison between studies and assessment of study quality. Moreover, studies to date using mobile eye-tracking during face-to-face interaction generally have relatively small sample sizes (Franchak et al., 2018; Honma, 2013; King et al., 2013; Macdonald & Tatler, 2018; Spezio et al., 2007; Vabalas & Freeth, 2016; Z. Ye et al., 2012). This may be because analysis of these mobile eye-tracking data so far has been labour-intensive and not straightforward to automate.

Areas-of-interest are commonly used in eye-tracking data to assess how often and how long participants fixate their gaze within a certain area – for instance, another person’s face or a specific part thereof. This information is used to infer the level of gaze on the eyes (Horley, Williams, Gonsalvez, & Gordon, 2003). Based on such analyses, researchers have for example concluded that individuals with social phobia avoided looking at facial features, in particular the eyes, compared to controls (Horley et al., 2003). Whereas for screen eye-tracking several methodologies have been developed to automatically generate areas-of-interest (Chawarska, Shic, & disorders, 2009; Hessels, Benjamins, Cornelissen, & Hooge, 2018; Hunnius & Geuze, 2004), creating areas-of-interest for wearable eye-tracking videos is more challenging (R. S. Hessels et al., 2018). Because mobile eye-tracking glasses offer freedom of movement and can be used “in the wild” (De Beugher, Brône, & Goedemé, 2016), they lack a fixed reference frame (as in screen-based eye-tracking). Thus far researchers have mostly manually annotated areas-of-interest in wearable eye-tracking videos for each video frame – i.e. around 25 times per second of video material (depending on characteristics of the eye-tracking device) (Franchak et al., 2018; Garrido-Jurado, Munoz-Salinas, Madrid-Cuevas, & Medina-Carnicer, 2016; R. S. Hessels et al., 2018). However, the manual creation of these areas-of-interest makes this process vulnerable to subjective interpretations which can negatively influence the reliability of the areas-of-interest identification. In addition, this process is extremely labour-intensive and thus time-consuming (R. S. Hessels et al., 2018). To summarize, small sample sizes, subjective interpretations and labour-intensive analysis are downsides of manual creation of areas-of-interest.

; Computer vision algorithms may reduce the complexity and subjectivity of mobile eye-tracking data analysis of face-to-face interactions (Callemein, Van Beeck, Brône, & Goedemé, 2018; De Beugher, Brône, & Goedemé, 2014; Duchowski et al., 2019). Computer vision algorithms are able to construct areas-of-interest through automatic detection of human body parts such as faces, torsos, or hands in dynamic videos (Callemein et al., 2018; Duchowski et al., 2019). Using mobile eye-tracking data in combination with computer vision algorithms could enable researchers to automatically identify when, and how long, individuals fixate their gaze on specific areas of other people. However, studies so far that used algorithms to analyse eye-tracking data either report limited analytic details (Honma, 2013; Spezio et al., 2007), or may be using sub-optimal algorithms (Duchowski et al., 2019; Z. Ye et al., 2012). Ye and colleagues documented an accuracy of 73% on one video of 7 min when comparing the algorithm to the ground truth (Z. Ye et al., 2012). Duchowski and colleagues automatically analysed five laboratory eye-tracking sessions of less than 30 s with good light and little movement (Duchowski et al., 2019). They reported an accuracy ranging from 9.6% (mouth) to 99.5% (left eye) for the different facial features, when comparing the instructed (expected) gaze time (100%) to the measured gaze time within an area-of-interest. Although these authors concluded that their algorithm was successful, we believe it can be improved. Because wearable eye-tracking data often involves highly mobile (blurry) and low-quality footage, specific areas-of-interest (e.g. the face) can be supplemented with full-body detection and specific techniques to distinguish between different people in an interaction (Bashbaghi, Granger, Sabourin, & Parchami, 2019). These specific detection techniques can be used to automatically identify areas-of-interest around faces, thus reducing the complexity of analysis of wearable eye-tracking data. Callemein and colleagues compared two publicly available frameworks for head detection, YOLOv2 and OpenPose, and found that the average precision is 57% for YOLOv2 and 72% for OpenPose when comparing the automatic analyses to an existing dataset (INRIA) (Callemein et al., 2018; Dalal and Triggs, 2005). The authors concluded that OpenPose outperformed the YOLOv2 model. Using computer vision algorithms to identify areas-of-interest may offer a more reliable, accurate and quick method as compared to manual annotations. However, it is currently unclear how computer vision algorithms perform compared to manual analysis.

Therefore, the aim of this study is to compare gaze-to-face levels identified by a computer vision algorithm to those identified by human annotators on mobile eye-tracking data in interpersonal interactions using an area-of-interest. The results of this study can be used to improve the state of the art of assessing gaze direction in face-to-face human interactions.

## Methods

### Design

A computer vision algorithm and three human annotators (AM, LO, and TB) annotated mobile eye-tracking data, to assess interrater variability between the computer algorithm and the human annotators. Data for the present analyses were collected as part of a larger prospective observational study, designed to assess the effect of eye contact between physicians and their patients on the patient–physician relationship. Internal medicine residents in an out-patient clinic wore a wearable eye-tracker (Tobii Pro Glasses 2) during regular follow-up consultations with patients (*N* = 100) (Tobii Pro AB, Stockholm, Sweden). Consultations were additionally recorded on camera and all participants responded to questionnaires before and after the consultation. Data collection started in February 2018 and ended in May 2019. The study was exempted from the Medical Research Regulations Involving Human Subjects Act by the Medical Ethics Committee of the Amsterdam University Medical Centres, location AMC. Patients and residents gave written informed consent.

### Procedure

**Fig. 1:**
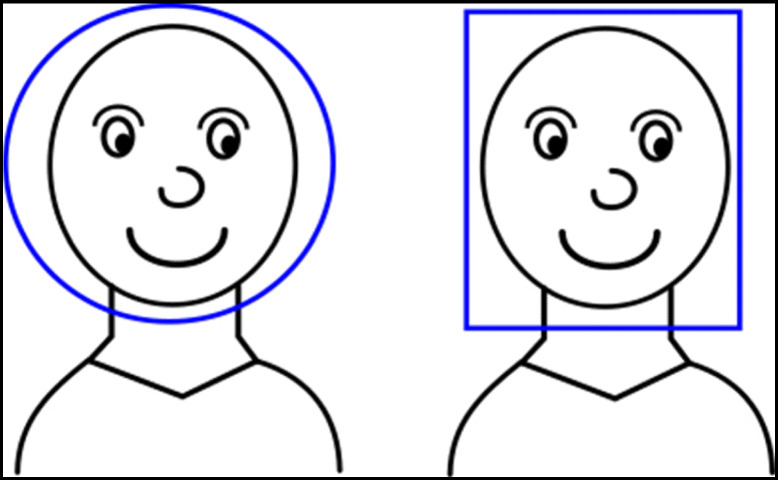
An illustration of the primary area-of-interest (*right*) and the explorative area-of-interest (*left*)

First, for the primary analysis we performed manual and computer vision algorithm analyses on the first minute of seven eye-tracking videos. One complete video (#6) was annotated by two annotators to assess the interrater reliability between manual annotations. Second, we additionally conducted explorative analyses on two videos (#6 and #7), to test the robustness of the computer vision algorithm when using lengthy eye-tracking videos (of which one (#7) included an additional interactor) and different area-of-interest shapes. The default shape of the area-of-interest by our algorithm is rectangular. However, an oval area-of-interest might better approximate the shape of a face by reducing the degree of overestimation of face-gaze (in the angles). Therefore, we assessed the difference between a rectangular and an oval area-of-interest by comparing a manual oval area-of-interest shape to the automatically created rectangular area-of-interest. We refer to Fig. 1 for an illustration of the area-of-interest shapes. We have chosen large areas-of-interest around the face over smaller areas-of-interest around the eye region, because overall large areas-of-interest are more noise-robust compared to smaller areas-of-interest (Hessels, Kemner, van den Boomen, & Hooge, 2016).

### Participants and eye-tracking videos

We randomly selected seven eye-tracking videos of consultations between patients and physicians. This limited number of videos was chosen due to the time- and labour intensity of the manual annotations. Patients that participated in our study (*N* = 7) were on average 61 years old (range, 41–77 years) and four were female. Physicians (*N* = 7) were on average 35 years old (range, 33–38 years) and four were female. Visual acuity was normal or corrected to normal for all physicians. No eye-tracking data needed to be discarded because of data loss.

Beforehand, it was tested whether their eyes were suitable for a sufficient calibration quality as indicated by our eye-tracking software (the Tobii Pro Glasses Controller software). Calibration was done by having the participant’s gaze focus on a specific calibration target (black dot on a calibration card) at 0.75–1.25-m distance. The calibration ensures the accuracy of the measurement of the eye-tracking glasses and sufficient calibration quality is a prerequisite for collecting precise eye-tracking data (Nyström, Andersson, Holmqvist, & Van De Weijer, 2013). A recent study shows that the calibration quality of the mobile eye-tracking equipment we used remains of good quality even when suffering from slippage (Niehorster et al., 2020).

**Fig. 2:**
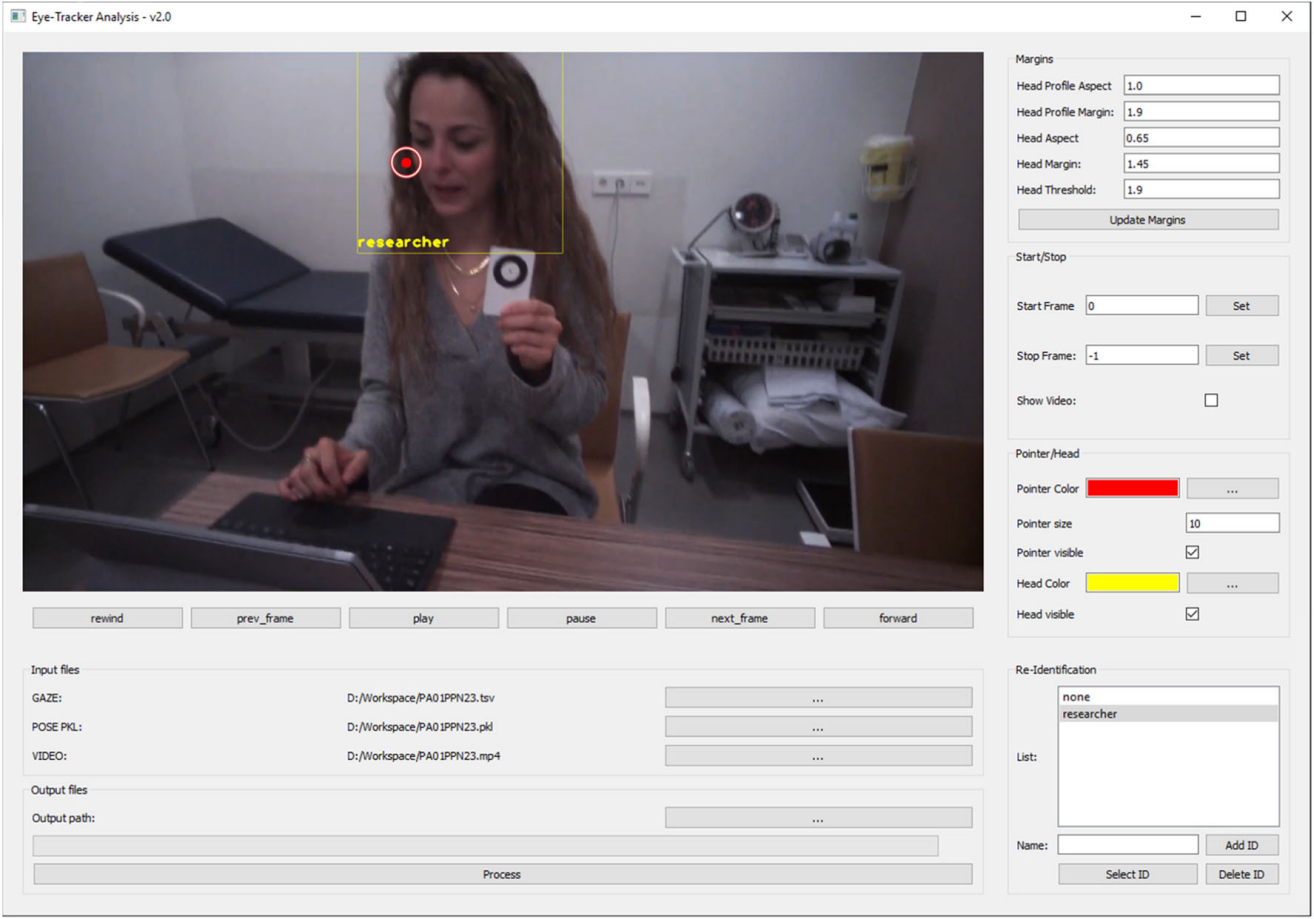
A screenshot of the eye-tracker analysis software in operation

All physicians wore the eye-tracking glasses throughout the consultation. All recordings started from the moment the physician opened the door of the consultation room and invited the patient (and caregiver) in. Next, the physician and patient (and caregiver) sat down on either side of a desk. Occasionally a physical examination took place (see Appendix). A screenshot of the physician’s outlook can be seen in Fig. 2.

### Manual analysis

All manual analyses were performed by three human annotators using Tobii Pro Lab Analyzer software for eye-tracking analysis. This software displays the forward-looking video camera frames (located on the nose bridge and capturing the environment the person is looking at). Annotators can manually draw an area-of-interest on each video frame (25 times per second), using an area-of-interest tool (Tobii Pro AB, Stockholm, Sweden). The gaze location is not shown on screen while drawing the area-of-interest. Annotators judge whether the face of an individual is displayed in the video image and create one or multiple areas-of-interest. The annotators were instructed to draw the area-of-interest around the face, similar to the areas-of-interest shown in Fig. 1. Both the rectangular and the oval drawn area-of-interest should capture the whole face including a small margin around it. The annotators were able to drag the area-of-interest from frame to frame, although each movement of the person wearing the eye-tracker and the interactors shown on screen demanded manual adjustment of the previously set area-of-interest. When two areas-of-interest are present in the frames (e.g. both a patient and a caregiver) this doubles the manual workload. The size and rotation of the area-of-interest need to be manually adjusted depending on the position in the frame. The time it takes to analyse a video of 1 min depends on the annotator’s characteristics, we estimate it to be around 50 min. The output of the manual analysis is a spreadsheet indicating per video frame (40 ms) whether or not the physician’s gaze was focused within the face (the area-of-interest). This was indicated by a value of ‘1’ when the gaze matched the face-area, and a value of ‘0’ when it did not. All manual analyses were displayed on video and the areas-of-interest were visually checked for shape, size and location accuracy by the first author (CJ) to assure that the manual analysis would suffice as ground truth. No major empirical errors were detected. Videos #1 to #5 were all coded once by one annotator each, video #6 was coded twice by a single annotator (the second time using a different area-of-interest size) and once by a different annotator, and video #7 was coded twice by the same annotator (the second time using a different area-of-interest size). We have randomly chosen video #6 to be double coded. For an overview of annotators, videos, numbers of frames, area-of-interest shape and number of individuals shown in the videos we refer to our Appendix.

### Computer vision analysis

For the computer vision analyses we first we performed a single class automated analysis, indicating whether the gaze was located on any face (resulting in a ‘1’ on the spreadsheet) or not (resulting in a ‘0’ on the spreadsheet). Second, we performed a multiple class automated analysis indicating whether the gaze was located on a specific individual’s face resulting in a ‘1’ when it was located on for example the patient’s/caregiver’s/researcher’s face, and a ‘0’ when not. The output was specified per 40 ms.

Using the computer vision annotation requires less manual work. The algorithm performs the calculations based on the processor speed. The algorithm processes a video of 1 min in less than 1 min, when using a NVIDIA 1080 Ti GPU. Therefore, using the computer vision software for analyses requires less time than the manual analyses.

**Fig. 3:**
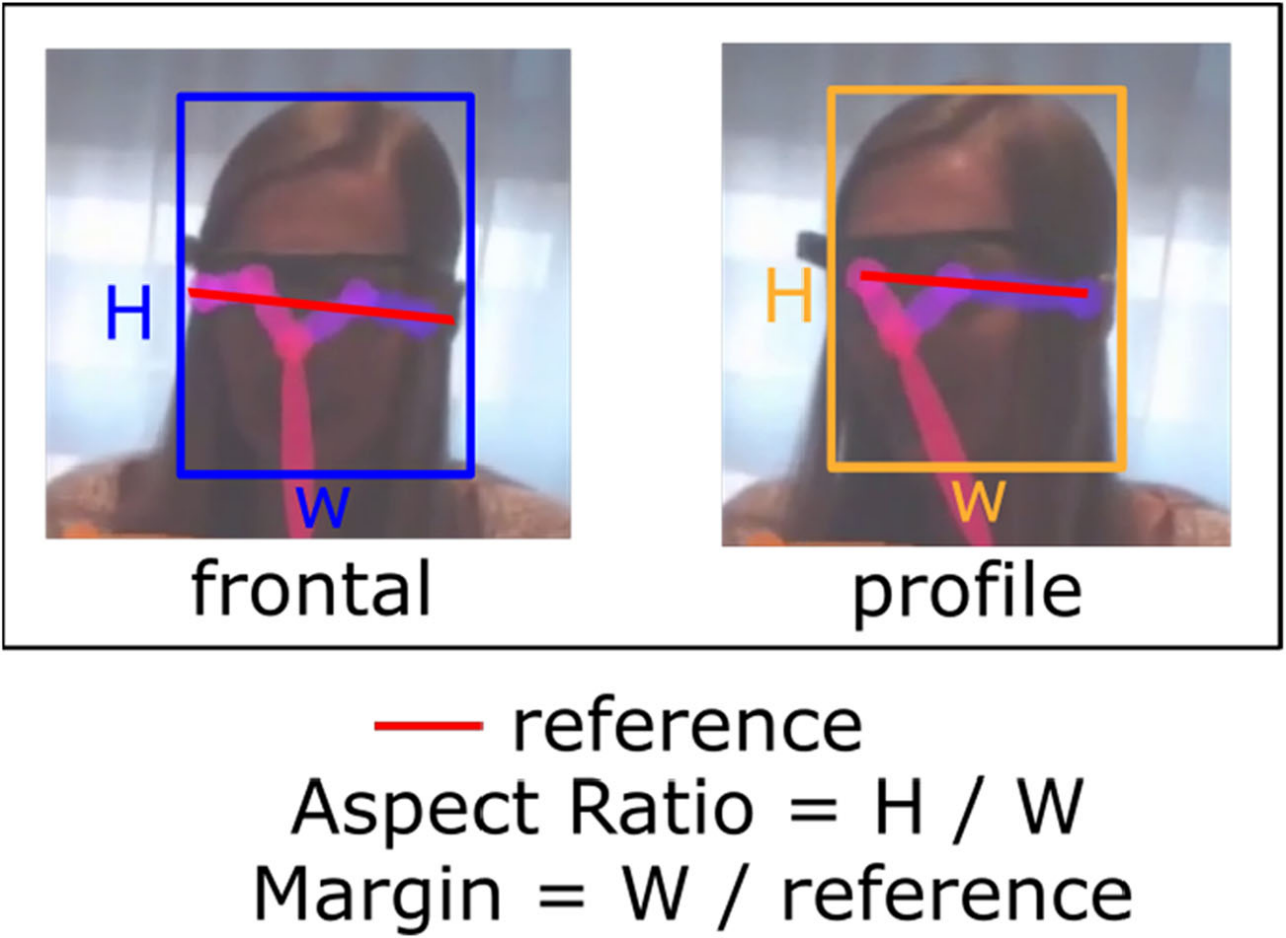
A graphical example of how the aspect ratios and margin scales for frontal and profile faces were determined

The computer vision analysis was operated using software specifically designed for this study which we coined ‘Eye Tracker Analysis’. See Fig. 2 for a screenshot of eye-tracker analysis operating on wearable eye-tracking data. The software is available and can be downloaded following this link: https://osf.io/4uy35/?view_only=785a011774cf4c4f8c5e4608b34a2a38. To operate the software, we used raw data, i.e. the wearable eye-tracking video and the gaze location (i.e. the gaze screen coordinates) without any fixation or attention filter (Tobii Pro, 2019b). Beware that all data were synchronised (eye-tracking video and gaze location), which we were able to verify in the video display, as shown in Fig. 2. The area-of-interest was calculated using parameters (aspect and profile ratio) as illustrated in Fig. 3. These parameters can be either kept constant or manually adjusted to a bigger or smaller size when desired, making the software semi-automatic. In our approach, we first calculated the mean parameters of the manual annotations and used these to create the areas-of-interest. The software offers default parameters, based on our calculations, but these can be adjusted if desired. For a detailed description of the technique of the computer vision algorithm, see Box 1.

*Box 1.* A detailed description of the techniques used in the computer vision analysis.

**Table:** 

To calculate the areas-of-interest we used an OpenPose base head detection based on a previously published study (Callemein et al., 2018; Cao, Hidalgo, Simon, Wei, & Sheikh, 2018). The OpenPose framework detects 18 anatomical key-points in the images that together represent the full human pose skeleton. In the present study, only the five key-points located in the head region, comprising the location of the nose and both ears and eyes, were used (see Fig. 3 for an illustration). Whenever two or more of these key-points are visible the head region is identified, when only one key-point is visible, the head remains undetected. Defining a bounding box around these points creates a rectangular area-of-interest. This bounding box enables a dynamic and autonomous definition of the head area-of-interest around each person’s face visible in the image. In most cases, a simple bounding box around these points would not suffice, since the full head region is not covered by these five points. This issue is solved using the relative distance and orientation of these points to first determine the face orientation (Callemein et al., 2018). We defined a frontal face when all five face points were available, or a profile face when fewer points were visible due to for example turning of the face. Using the largest distance between the available points as the area-of-interest width in pixels, we calculated the area-of-interest height by multiplying the width with the aspect ratio parameter. To ensure the complete coverage of the face, we also used an additional scale (margin) parameter. Different parameters are needed depending on whether the image shows a frontal or a profile face and this accounts for variation in centre location for the area-of-interest. The areas-of-interest are thus calculated based on these parameters.	

The single class algorithm is not able to distinguish between different people participating in an interaction. To address this issue, we additionally used multiple class analysis with re-identification (Re-ID) techniques. This technique is able to recognize and re-identify individuals using specific characteristics of their appearance (De Feyter, Van Beeck, & Goedemé, 2018; He, Zhang, Ren, & Sun, 2016; Li, Zhao, Xiao, & Wang, 2014). In particular, we first used OpenPose to establish person detection (Cao et al., 2018; Cao, Simon, Wei, & Sheikh, 2017). We supplemented this detection with a ResNet artificial neural network architecture to produce a 128-dimensional description vector, called *embedding,* that is able to enclose the identity of a person using features such as colour and shape (De Feyter et al., 2018; He et al., 2016; Li et al., 2014). We used a ResNet-34 network trained on the CUHK03 dataset (the Chinese University of Hong Kong-03 dataset including 1467 identities) to extract such a deep face embedding for each upper-body area-of-interest of a person detected in the video. The *embeddings* for the upper-body poses were stored and used to compare each detected person in the videos against a gallery of labelled persons, using the Euclidean distance.

We saw that a few manually labelled occurrences of each person in this gallery suffice for each person appearing in the video to be automatically assigned to an identity. For most persons in the video, one labelled occurrence in the gallery suffices. However, if additional *embeddings* are added to the identity list throughout the video and frames, the Re-ID system improves throughout the analysis. Especially whenever wardrobe changes occurred, and therefore the embedding changed, we had to add the *embeddings* to the list accordingly, resolving this issue with only a limited effort. When no more than a single individual is present in the eye-tracking video, use of the multiple class Re-ID technique could be unnecessary. The multiple class Re-ID technique could also be used to adjust single class misclassifications.

### Agreement between manual and automated analyses

Cohen’s kappa (κ) (Cohen, 1986) was calculated to assess interrater reliability, i.e. agreement, between human annotators and the computer vision algorithm. A Cohen’s kappa of 1.0 indicates perfect agreement and a score of 0.0 indicates no agreement. Moreover, for all comparisons (both single class and multiple class) agreement was calculated using accuracy results. Accuracy results were calculated by comparing assessments frame-by-frame. Manual analysis was used as ground truth. Whenever assessment of the manual analysis matched the algorithm results this was classified as “true positive”. A “false positive” result occurred when the algorithm analysis did not match the manual analysis. For the multiple class analyses both the manual and the Re-ID analyses were labelled with identities. A “true-positive” result was when the gaze was located on a specific person according to both the algorithm and the manual analysis. A “false-positive” result in the multiple class analysis was similar to a “false positive” result in the single class analysis, and occurred when the algorithm did not match the manual analysis. Accuracy is then displayed in percentages of “true positives”. Furthermore, we used absolute normalized differences to illustrate the differences between the manual and the automated analyses. Absolute normalised differences were calculated using the absolute difference (the sum of both over and under identification) in seconds, divided by the total amount of time of the video. First, as a background check, we compared the two manual analyses on video #6. In case of sufficient agreement, we would proceed to compare the manual analyses of the seven videos to the computer vision algorithm. Second, for our primary aim we compared the manual annotations to both the single and to the multiple class analyses. Third, for our explorative aim we compared manual annotations of two longer videos (one involving an additional interactor) both with the regular and with a different shaped area-of-interest to the single- and multiple class automatic annotations.

## Results

### Primary aim: Comparing manual with automated analyses

The first minutes of all analysed seven videos, totalled 421.04 s of recorded data, comprising 10,526 frames. Overall, the manual annotators identified 204.48 s of face gaze vs. 212.40 s identified by the algorithm. The average absolute difference between the manual and the algorithm face-gaze annotations was 0.93 s (mean normalized absolute difference of 1.5%). Disagreements may be due to the set size of the parameters (which define the size of the areas-of-interest) in the algorithm. In the annotations performed by the algorithm, the size of the areas-of-interest is standard, whereas in the manual analysis it may vary.

As a background check, agreement between the manual analyses by two different human coders was calculated on one complete video (#6), resulting in a Cohen’s kappa of 0.99. Hence, we proceeded to compare the single class output of the manual analyses to the algorithm analyses (see Table 1). Results indicated high agreement scores (all κ ≥ .89) between the manual annotators and the algorithm.

**Table 1:** Single class agreement results on the first minute of all seven eye-tracking videos between manual annotators and the computer vision algorithm

Video	Duration (s)	Face-gaze duration (s)		Δ (s)	normalized Δ (%)	Cohen’s kappa
		Manual	Algorithm			
#1	59.84	30.44	30.60	– 0.16	0.27	0.97
#2	60.50	27.00	26.60	0.40	0.66	0.89
#3	60.04	30.08	31.40	– 1.32	0.22	0.94
#4	60.46	35.36	36.56	– 1.20	1.98	0.96
#5	60.20	24.80	26.00	– 1.20	1.99	0.98
#6	60.00	24.64	26.68	– 2.04	3.40	0.91
#7	60.00	34.4	34.56	– 0.16	0.27	0.90

Next, we compared the manual annotations and the multiple class output of the algorithm (see Table 2). The manual annotations identified in total 192.52 s of face-gaze on the patients, compared to 199.06 s for the algorithm. Overall, the average difference of the level of face-gaze between the manual annotations and the algorithm annotations was 0.92 s (absolute), normalized = 1.54%. The results displayed in Table 3 indicate high agreement scores of over (all κ ≥ .88) between the manual annotators and the algorithm.

**Table 2:** Multiple class identification accuracy and agreement results for all videos between human annotators and the computer vision algorithm using the Re-ID technique

Video	Duration of identity - manual (s)		Duration of identity – algorithm (s)		Δ (s)	Normalized Δ (%)	Cohen’s kappa	Re-ID accuracy (%)
	Patient *Caregiver*	Researchers	Patient *Caregiver*	Researchers				
#1	30.16	0.28	30.44	0.16	– 0.16	0.27	0.93	96.26
#2	26.90	0.10	26.5	0.10	0.40	0.66	0.88	94.18
#3	28.76	1.32	30.16	1.24	– 1.32	2.20	0.92	95.94
#4	34.20	1.16	35.72	0.84	– 1.20	1.98	0.93	96.50
#5	24.80	0.00	26.00	0.00	– 1.20	1.99	0.96	98.01
#6	24.56	0.08	26.56	0.12	– 2.04	3.40	0.91	95.80
#7	24.00 *10.36*	0.04	23.68 *10.80*	0.08	– 0.16	0.27	0.91	94.53

*Note:* Duration of identity: the time (in s) a specific individual is identified. The identities are patients and researchers, in video #7 additionally a caregiver is identified, the time (in s) the caregiver is identified is shown in italics

**Table 3:** Results of agreement analysis on videos #6 and #7 of long duration, and with a different shape of area-of-interest

Video, condition	Face-gaze duration (s)		Δ (s)	Normalized Δ (%)	Cohen’s kappa
	Manual	Algorithm			
#6, long duration, rectangular AOI	173.92	183.52	– 9.60	0.87	0.95
#6, long duration, oval AOI	174.6	183.52	– 8.92	0.81	0.95
#7, long duration, rectangular AOI	229.16	239.4	– 10.24	1.35	0.89
#7, long duration, oval AOI	209.24	239.4	– 30.16	3.97	0.87

For the Re-ID confusion matrices, indicating the accuracy of the person identification of the algorithm compared to the human annotations, see Appendix. All confusion matrices show a limited amount of confusion between the different identities (researchers, patients, and caregiver). Most confusion can be accounted for by the single class annotations.

### Explorative aims: Testing the robustness of the computer algorithm

We had explorative aims comparing the workings of the algorithm to empirical challenges. We compared the algorithm on manual annotations of two longer videos (one with an additional interactor) and the same videos with a different area-of-interest shape (oval), using the single class algorithm and the multiple class algorithm. Video #6 had a duration of 1102.76 s (27569 frames) and video #7 had a duration of 758.92 s (18,973 frames). In video #6 the manually identified face-gaze was 173.92 s with a rectangular area-of-interest and 174.6 s with an oval area-of-interest, versus the algorithm that identified 183.52 s of face-gaze. In video #7 the manually identified face-gaze was 229.16 s with a rectangular area-of-interest and 209.24 s with an oval area-of-interest. The algorithm identified 239.4 s of face-gaze. We refer to Table 3 for our agreement analyses with the single class output and to Table 4 for our agreement analyses with the multiple class output. In all explorative conditions, the Cohen’s kappa values remain above κ ≥ .85.

**Table 4:** Identification accuracy and agreement results for the explorative videos between human annotators and the computer vision algorithm using the Re-ID technique

Video, condition	Duration of identity – manual (s)		Duration of identity – algorithm (s)		Δ (s)	Normalized Δ (%)	Cohen’s kappa	Re-ID Accuracy (%)
	Patient *Caregiver*	Researchers	Patient *Caregiver*	Researchers				
#6 long duration, rectangular AOI	173.84	0.08	183.4	0.12	– 9.52	0.86	0.95	98.61
#6 long duration, oval AOI	173.56	0.04	183.4	0.12	– 9.76	0.88	0.95	98.65
#7 long duration, rectangular AOI	195.6 *33.52*	0.04	189.72 *49.16*	0.52	22	2.90	0.87	93.92
#7 long duration, oval AOI	182.16 *26.88*	0.2	189.72 *49.16*	0.52	15.04	1.98	0.85	93.18

*Note:* Duration of identity: the time (in s) a specific individual is identified. The identities are patients and researchers, in video #7 additionally a caregiver is identified, the time (in s) the caregiver is identified is shown in italics

## Discussion

The primary aim of this study was to test whether gaze-to-face levels identified by a computer vision algorithm are comparable to those identified by human annotators on mobile eye-tracking data using areas-of-interest. For our primary aim, our results show high interrater agreements between the human annotators and the algorithm, with Cohen’s kappa ranging from 0.88 to 0.98 and absolute normalized differences of less than 2%. The Re-ID algorithm can help to distinguish different individuals when there are two people visible on the eye-tracking data (with an accuracy of > 94% compared to the human annotators). Moreover, for our exploratory aims we found that the algorithm performed well compared to manual analyses in variable conditions, such as when using long videos, different area-of-interest shapes or when analysing videos involving an additional interactor. Our results indicate that computer analyses may be used as an alternative to manual analyses on mobile eye-tracking data.

The algorithm used in this study automatically identifies area-of-interest, i.e. human faces “in-the-wild” (De Beugher et al., 2016), which is an advancement on the software currently available, which does not automatically generate areas-of-interest on moving objects (Tobii Pro, 2019b). Previous software aimed at face identification has limitations: it is only suitable for data acquired in good conditions, e.g. with sufficient lighting, an acceptable distance between individuals and no background noise (Duchowski et al., 2019). Our software, based on previous work by Callemein et al. uses full body detection to identify the heads of individuals (Callemein et al., 2018; Cao et al., 2018; Redmon & Farhadi, 2017). Using full-body detection, the algorithm can identify faces even when the targeted individuals tilt or yaw their heads. Furthermore, compared to previous studies, we analysed more, longer, and more variable videos including movement, different light conditions and more individuals. Possible downsides of our approach may be that it only identifies faces of individuals, while manual annotations may be extended to objects.

Until present, “in-the-wild” technologies for studying communication often used manual annotation and were unable to distinguish between different individuals in the video (Calvitti et al., 2017; Farber et al., 2015; Street et al., 2014). The latter is needed to provide a more detailed analysis of specific communication styles in different (applied) settings and involving several participants. Moreover, the algorithm is able to provide additional meta data that could be useful for future analysis, such as the location of the head, whether a specific person is present and how many people are visible (Callemein et al., 2018). Our results are preliminary and show that even in non-ideal situations, e.g. when using highly mobile and blurry footage, head detection is still comparable to detection by human annotators. In the future, it would be interesting to investigate the exact frames where the manual analysis and the algorithm analysis differ. This would create more valuable understanding about the limitations of each analysis method.

Analyses by a computer vision algorithm are not subject to annotator fatigue or subjectivity, which may increase reliability, although this needs to be further tested. The algorithm performs consistently whereas humans are subject to uncontrollable external factors. The use of the software is considerably more efficient compared to manual analysis. Furthermore, when using the software, visual inspection of the area-of-interest is possible in the representation of the eye-tracking video on screen. The face-gaze can be checked frame-by-frame. Therefore, researchers using this algorithm can check (and possibly correct) the accuracy of the detected face-gaze, such that a fully accurate analysis can be performed with much less manual labour.

A limitation of our study is the relatively small sample size (*N* = 7 videos). However, the data that we used, resulted in 100,613 frames. A comparison between the algorithm and a manual annotator is made for each of these frames. Moreover, we tested the robustness of the algorithms by posing additional challenges and using extremely mobile eye-tracking data, including in difficult indoor lighting conditions. Furthermore, the underlying techniques that we applied in this setting have previously been tested on very large datasets (Callemein et al., 2018; Cao et al., 2018; Cao et al., 2017; Simon, Joo, Matthews, & Sheikh, 2017; Wei, Ramakrishna, Kanade, & Sheikh, 2016), where they have proven their effectiveness. A second limitation is that we have not tested the algorithm in videos involving more than four people where other interactors are extensively visible on screen. Whether the algorithm can successfully and systematically distinguish between three or more different people remains to be verified. At present, we may conclude that our software is most suitable for dyadic face-to-face interactions.

Very recent technologies offer even more refined detection compared to the technology used in the present study (Alp Güler, Neverova, & Kokkinos, 2018). Such technologies would for instance enable detecting refined head-shaped areas-of-interest (He et al., 2017 ; Bolya, Zhou, Xiao, & Lee, 2019a, 2019b). However, our results indicate, in line with previous findings, that the size of the area-of-interest makes only a slight difference for the detected amount of face-gaze (Hessels et al., 2018). Previous comparisons between different area-of-interest sizes were based on screen eye-tracking and compared different areas-of-interest sizes for facial features (R. S. Hessels et al., 2018).

The software is available and could be used to research eye contact in real-world or observational settings. Whereas the algorithm is able to unilaterally detect the frequency and duration of people’s gaze towards the face of the interactor, future research could assess whether the interlocutor is gazing back, using gaze-locking data (Smith, Yin, Feiner, & Nayar, 2013). This technique detects whether the eyes of the interlocutor are faced towards the eye-tracking camera. However, to date, mobile eye-tracking videos are too low in resolution to enable measuring mutual eye contact.

Concluding, we have found that algorithm analyses of face-gaze using areas-of-interest are comparable to face-gaze of manually annotated areas-of-interest. Therefore, manual analyses of eye-tracking videos can be replaced or supported by software. The algorithm we presented here can automatically detect faces in mobile eye-tracking videos and accurately create areas-of-interest to assess face-gaze. Furthermore, the algorithm can distinguish between different individuals. This is an advancement of the state-of-the-art analysis in mobile eye-tracking research.

## Appendix

### Overview of annotators, videos, numbers of frames and area-of-interest shape and number of people shown in the video

**Table:**

Annotator	Video	Frames	Area-of-interest shape	No. of people in video	Physical examination
AM	1	1496	rectangular	2	No
AM	2	1513	rectangular	2	No
AM	3	1501	rectangular	2	No
AM	4	1514	rectangular	3	No
AM	5	1505	rectangular	**1**	No
LO	6	27569	rectangular	**1**	No
LO	6	27569	oval	1	No
TB	6	27569	rectangular	1	No
TB	7	18973	rectangular	3	Yes
TB	7	18973	oval	3	Yes

### Confusion matrices of the multiple class output for comparison between the manual and algorithm annotations

Video #1

**Table:**

		algorithm		
		none	patient	researcher
manual	none	**93.55**	6.33	0.11
	patient	0.98	**99.02**	0
	researcher	0	0	**100**

Video #2

**Table:**

		algorithm		
		none	patient	researcher
manual	none	**96.92**	3.95	0.14
	patient	2.92	**97.08**	0
	researcher	**57.14**	0	**42.86**

Video #3

**Table:**

		algorithm		
		none	patient	researcher
manual	none	**95.35**	4.65	0
	patient	7.28	**92.72**	0
	researcher	0	0	**100**

Video #4

**Table:**

		algorithm			
		none	patient	researcher1	researcher2
manual	none	**93.72**	0	0	6.28
	patient	16.67	**83.33**	0	0
	researcher1	3.7	0	**96.3**	0
	researcher2	1.67	0	0	**98.33**

Video #5

**Table:**

		algorithm				
		none	patient	researcher1	researcher2	researcher3
manual	none	**93.49**	6.51	0	0	0
	patient	0.12	**99.65**	0	0.23	0
	researcher1	10	0	**75**	15	0
	researcher2	0	0	0	0	0
	researcher3	33.33	0	0	0	**66.67**

Video #6

**Table:**

		algorithm	
		none	patient
manual	none	**96.61**	3.39
	patient	0	**100**

Video #7

**Table:**

		algorithm			
		none	patient	caregiver	researcher
manual	none	**93.75**	2.81	3.44	0
	patient	4.67	**95**	0	0.33
	caregiver	3.09	1.16	**95.75**	0
	researcher	0	**100**	0	0
